# Excimer Laser Surgery: Biometrical Iris Eye Recognition with Cyclorotational Control Eye Tracker System

**DOI:** 10.3390/s17061211

**Published:** 2017-05-25

**Authors:** Bojan Pajic, Zeljka Cvejic, Zoran Mijatovic, Dragan Indjin, Joerg Mueller

**Affiliations:** 1 Swiss Eye Research Foundation, Eye Clinic ORASIS, 5734 Reinach AG, Switzerland; bpajic@datacomm.ch (B.P.); joerg.mueller@nova-optik.ch (J.M.); 2 Faculty of Sciences, Department of Physics, University of Novi Sad, Trg Dositeja Obradovica 4, Novi Sad 21000, Serbia; zeljkac@uns.ac.rs; 3 Medical Faculty, Military Medical Academy, University of Defans Belgrade, Belgrade 11000, Serbia; 4 Division of Ophthalmology, Department of Clinical Neurosciences, Geneva University Hospitals, Genève 1205, Switzerland; 5 School of Electronic and Electrical Engineering, University of Leeds, Leeds LS2 9JT, UK; d.indjin@leeds.ac.uk

**Keywords:** cyclorotation control eye tracker system, Excimer laser, refractive surgery

## Abstract

A prospective comparative study assessing the importance of the intra-operative dynamic rotational tracking—especially in the treatment of astigmatisms in corneal refractive Excimer laser correction—concerning clinical outcomes is presented. The cyclotorsion from upright to supine position was measured using iris image comparison. The Group 1 of patients was additionally treated with cyclorotational control and Group 2 only with X-Y control. Significant differences were observed between the groups regarding the mean postoperative cylinder refraction (*p* < 0.05). The mean cyclotorsion can be calculated to 3.75° with a standard deviation of 3.1°. The total range of torsion was from −14.9° to +12.6°. Re-treatment rate was 2.2% in Group 1 and 8.2% in Group 2, which is highly significant (*p* < 0.01). The investigation confirms that the dynamic rotational tracking system used for LASIK results in highly predictable refraction quality with significantly less postoperative re-treatments.

## 1. Introduction

In recent years, several improvements in the diagnostic aspects of refractive corneal surgery have been introduced which have contributed to improved precision and the availability of new clinical information such as wavefront aberration maps and three-dimensional corneal maps with high precision elevation data [1,2].

From the indication and algorithm points of view, parallel developments have been started by expanding the treatment ranges for high levels of astigmatism, aspheric ablation profiles, and the correction of high order aberrations [3,4,5,6].

These developments have resulted in the therapeutic components of a refractive platform that needs to be equipped with new technologies in order to provide a laser system capable of delivering superior precision and to utilize a new level of available information. It is well known that the eye can rotate several degrees when a person moves from a sitting position to a prone position. Having in mind that wavefront and topographic data are collected in the sitting position and that refractive surgery is performed in supine position, there is a great chance for potential error. Therefore, if the laser is tracking the center of the pupil, it would be most impossible to detect a rotational change because the center of the pupil may not be affected.

One of the essential components required to achieve this goal is a sophisticated eye tracking technology. The automated system that utilizes an eye tracking feature is a part of today’s novel refractive laser systems. 

Technolas 217z100P eye tracking system is a video-based system that uses infrared (IR) radiation to illuminate and capture the image by combining the advantages of being independent of the surrounding iris color conditions and the illumination level of the surgical field.

The relevance of cyclotorsion and its impact on the effectiveness of the ablation process are well known and several other systems have begun to include compensating technologies into their platforms [7]. As the observed rotational misalignment during the ablation cannot be neglected, the eye tracking system could improve surface ablation results when treating high astigmatism or when being wavefront guided. 

## 2. Materials and Methods 

**Figure 1:**
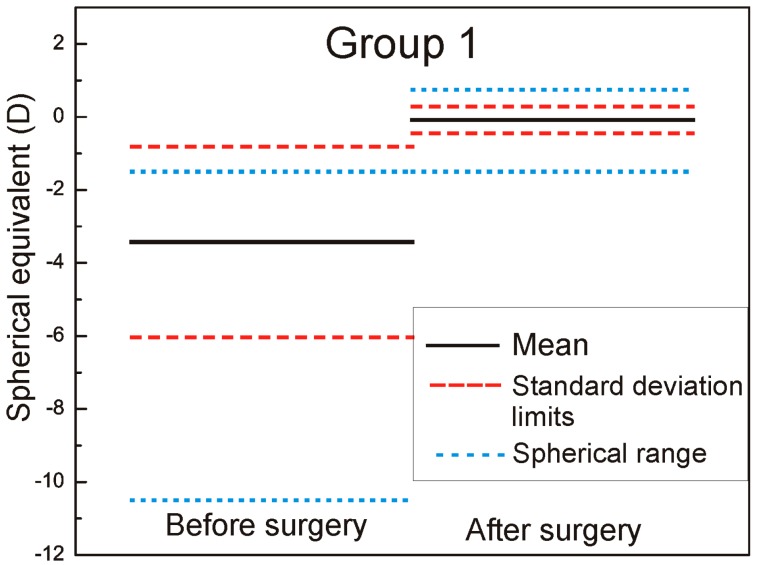
Refractive spherical equivalent pre- and postoperative Group 1.

**Figure 2:**
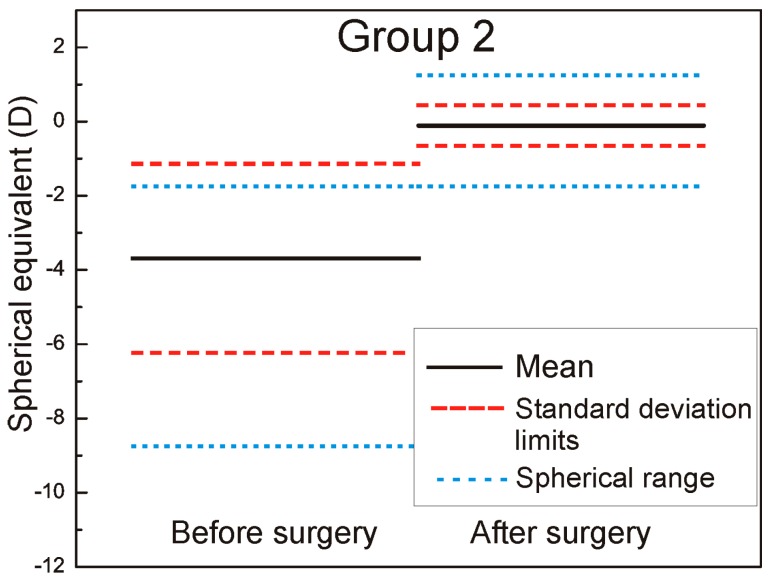
Refractive spherical equivalent pre- and postoperative Group 2.

We measured and adjusted the eye tracker system of Technolas 217z100P™ (Technolas, Munich, Germany) in LASIK (LASer-assisted In situ Keratomileusis) treatments on a total of 88 eyes in this study, 44 eyes with rotational tracker in Group 1 and 44 eyes with only X/Y tracker system in Group 2. For patients where bilateral treatment was performed, only data from the right eyes were used. The cyclotorsion from upright to supine position was measured using iris image comparison and it was not necessary to mark the cornea. All flaps created with the LDV high frequency femtosecond laser and energy range in nJ, were designed to deliver equivalent geometric dimensions 110 μm flap thickness, 9.5 mm flap diameter with superior hinge angle. The mean age of patients was: 36 ± 7.9 years (range: 23–61 years) in Group 1 and 36 ± 8.2 years (range: 21–62 years) in Group 2. The preoperative mean refractive spherical equivalent in Group 1 was −3.43 ± 2.61 D (range −10.5 to −0.75 D) and −3.69 ± 2.55 D in Group 2 (range −8.75 to −1.00 D) (the left part of the Figure 1 and Figure 2 marked as before surgery).

**Figure 3:**
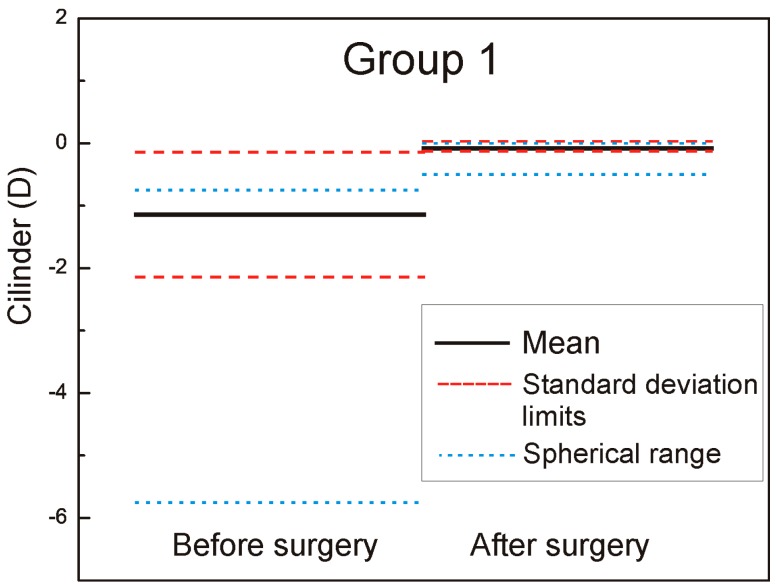
Mean cylinder pre- and postoperative Group 1.

**Figure 4:**
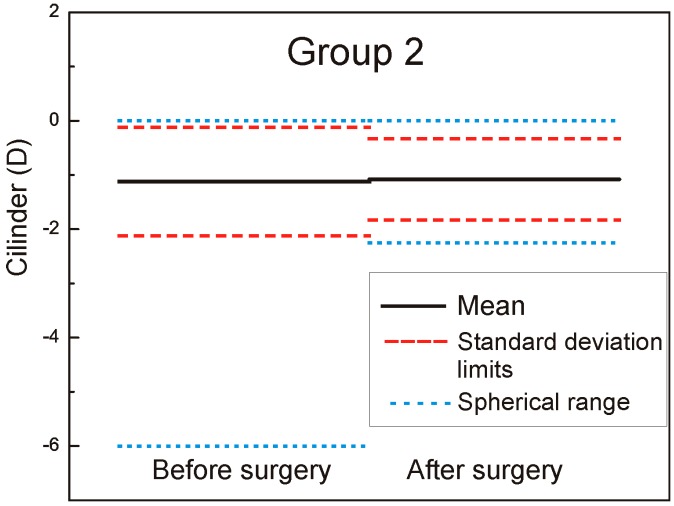
Mean cylinder pre- and postoperative Group 2.

The mean cylinder was −1.14 ± 1.00 D (range −5.75 to 0.00 D) in Group 1 and −1.12 ± −1.0 D (range −6.00 to −0.00 D) in Group 2 (the left part of the Figure 3 and Figure 4 marked as − before surgery). 

There was no significant difference between the groups regarding the mean preoperative spherical equivalent and cylinder refraction (*p* > 0.05).

**Figure 5:**
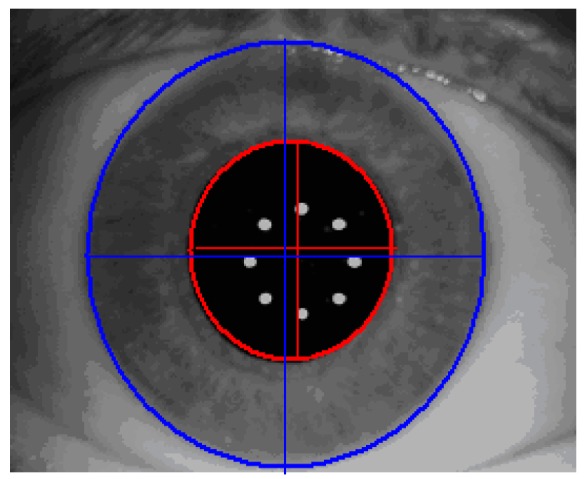
Pupil and limbus boundary recognition of the reference image.

By introducing the rotational eye tracker in the Technolas 217z100P™ the already approved X/Y tracker, which compensates actively for transversal movements of the eye, will be upgraded to compensate for misalignments in rotation. While it uses the acquired iris images only, additional objectively acquired information is needed to provide a rotational tracking capability. The 217z100P rotational tracker module is based on iris pattern recognition technology. It is well known that iris patterns are similar to finger prints with unique biometric properties of the individual. By using the appropriate technology, not only the individual eye can be recognized but also specific match parameters—such as rotational misalignment between two images of the same iris—can be obtained. The basic data used to analyze iris patterns are acquired infrared images of the iris. With special image processing and normalization technologies, the iris images are processed to account for differences in illumination conditions (large and small pupils) or even from different systems (such as Zywave wavefront sensor and laser system). The Zywave wavefront sensor is a diagnostic system which utilizes the same wavelength to acquire an Infrared (IR) image forms, the eye, and the corresponding iris [8]. Finally, the process described above results in a digital code which is created as follows: determine automatically pupil boundary and limbus boundary of the reference image (Figure 5).

**Figure 6:**
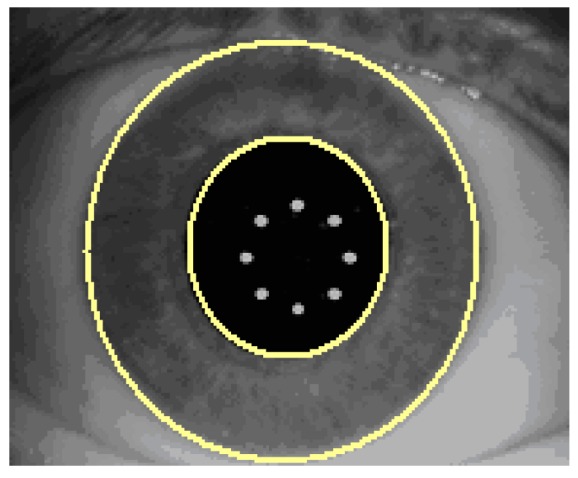
Visible iris pattern definition.

Certain sanity checks are applied to ensure that the automatically identified pupil and limbus geometries are within reasonable measures. The visible iris pattern defined as the structures between the pupil boundary and the limbus edge are used to create a normalized band (Figure 6). 

**Figure 7:**
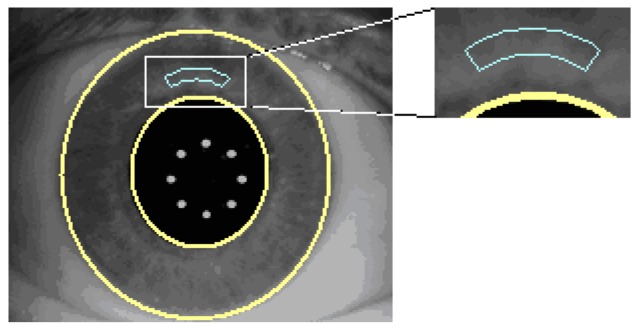
Digital iris code performance.

All operations which lead to the final digital iris code are performed within this normalized band (Figure 7). 

Within this band of iris image data, a unique digital code is generated. The basic data stream of this digital code consists of information obtained at 980 different iris locations. This iris code is, therefore, a unique code for this specific iris pattern and is used to detect potential torsional deviations between two different images from the same iris. The output parameters of the rotational eye tracker system—such as pupil radius/center and limbus radius/center—are given in resolution less than 30 µm. The rotation angle between images is within ±14.8° and with the resolution of 0.7° (=360°/512). Response time is 40 ms (i.e., 25 Hz).

During the static cyclotorsion compensation, the first step is a diagnostic phase which consists of obtaining IR iris image and deriving digital iris code from this reference iris image. This is followed with a planning phase where obtained data are transferred to the laser system. The next phase is a cyclotorsion compensation phase in which the patient’s head is aligned in the supine position. After that, the second IR iris image is acquired for comparison between the reference and actual iris code. 

During to intra-operative rotational compensation phase (dynamic cyclotorsion compensation), the referenced image is used as the first image for providing the iris code. Afterwards, a new iris image and code are required, while laser system is treating the patient’s eye. The next step is determining rotational angle to achieve maximum overlap of iris code and transferring this info to the laser software. The correct treatment pattern is determined according to the rotational angle. 

To observe and track the eye movements of a patient during surgery, the laser is equipped with two different cameras for the eye tracking system. One camera detects eye movements in the x/y direction. It is optimized for the area around the pupil and operational frequency is 240 Hz. The other eye-tracker camera detects the rotational movements of the eye and has a wider display window, so that the iris and limbal region can be observed. Operational frequency of this camera is 25 Hz. The slower frequency of the rotational eye tracker camera is justified with the slow speed of cyclotoric movements of eyes, while the x/y tracker must handle very fast saccadic movements in the x/y direction.

Due to the different observation angles of the two camera systems, it is also possible to detect movements in the height of an eye (also called Z-tracking) by the observation of the pupil centers obtained from the both cameras. The Z-tracking is opposite to the lateral and torsional tracking of a passive tracking system which simply deactivates the laser application if the eye moves outside of a given range of ±0.5 mm of the nominal Z-position. It should also be mentioned that the lateral eye tracker system works independently from the other two systems (rotational and Z-tracking). All mentioned eye tracking modules can block a laser pulse if the position is not accurate. The surgeon is able to deactivate the rotational eye-tracker system (including Z-tracking) at any time of surgery, while the x/y tracker can remain activated. If it is needed, the user can manually deactivate the x/y tracker too.

The system delivers the cyclorotational angle from a supine to horizontal position and additionally the dynamic eye tracker systems records the angle differences during the surgery. This results in an overall angle from the beginning until the end of surgery. This value is taken for the mean calculation of all eyes where the surgeries were undertaken with the cyclorotational eye tracker system, referred to as Group 1. 

## 3. Results

The six-month postoperative mean refractive spherical equivalent was −0.08 ± 0.36 D in Group 1 (range −1.5 to +0.75 D) and −0.11 ± 0.55 D in Group 2 (range −1.75 to +1.25 D) (the right part of the Figure 1 and Figure 2 marked as after surgery). In Figure 1 and Figure 2, it is very well revealed the refractive clinical outcome: preoperative vs. six months postoperative. 

**Figure 8:**
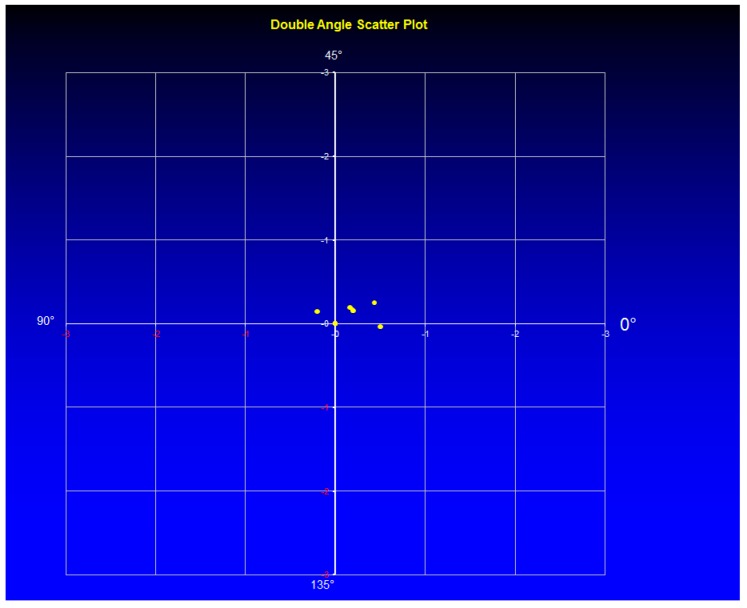
Double angle scatter plot for group 1.

**Figure 9:**
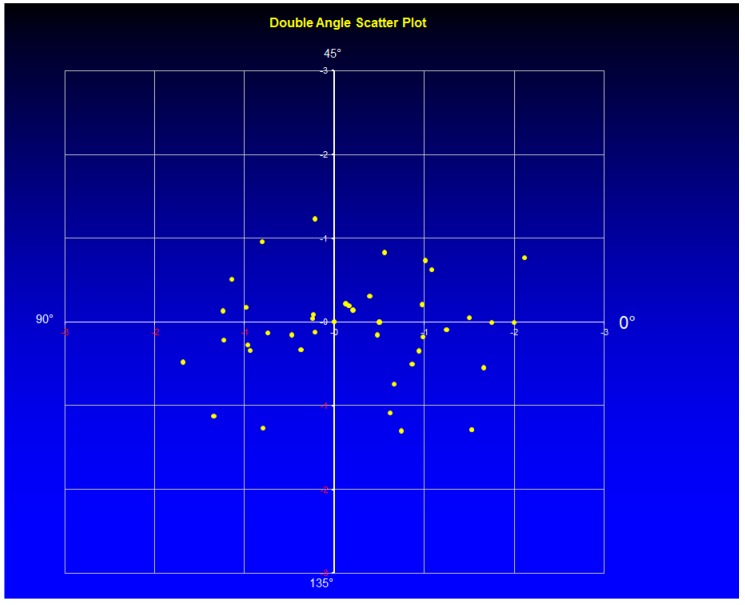
Double angle scatter plot for group 2.

The mean cylinder was −0.08 ± 0.05 D (range −0.5 to 0.0 D) in Group 1 and −1.08 ± 0.75 D (range −2.25 to 0.0 D) in Group 2 (the right part of the Figure 3 and Figure 4 marked as after surgery). In Figure 8, the double angle scatter plot of the cylinder value and angle is shown for the Group 1. Only a small range of astigmatism power and angle can be seen in contrast to the Group 2 where results for a double angle scatter plot analysis are shown in Figure 9. 

There is no much difference between the groups related to the mean postoperative spherical equivalent (*p* > 0.05) but significant difference in outcomes was observed related to the mean cylinder (*p* = 0.0043). The cylinder range of 2.25 D in Group 2 is remarkably high compared with the results obtained for Group 1, where only a range of 0.5 was measured. From supine to horizontal patient position there was an average static cyclotorsion deviation of −1.45° in Group 1. During the surgery the average cyclotorsion was calculated to be 3.75° with a standard deviation of 3.1°. Torsion was ranged from −14.9° to +12.6°. 

**Figure 10:**
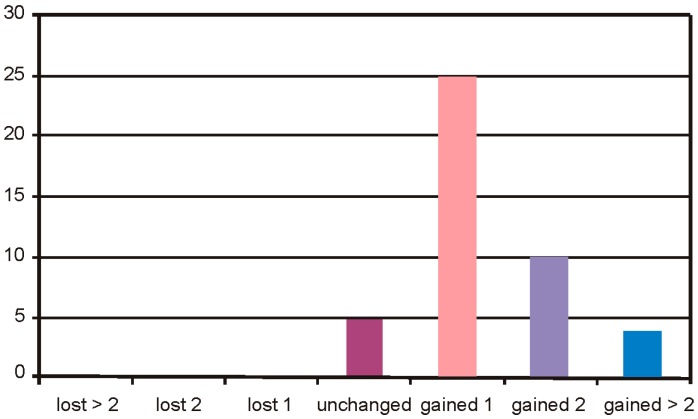
Treatment safety of Group 1.

In the Group 1, the safety of BSCVA (Best Spectacle-Corrected Visual Acuity) remained unchanged in 5 cases, 25 cases gained 1 line, 10 cases gained 2 lines, and 4 cases gained more than 2 lines of visual acuity (Figure 10). 

**Figure 11:**
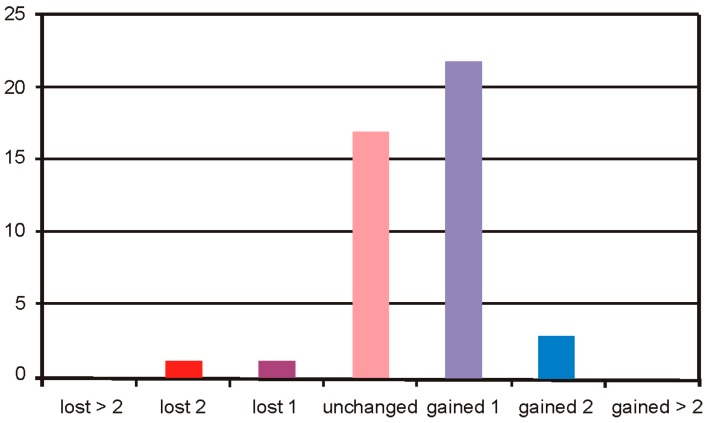
Treatment safety of Group 2.

In the Group 2, the safety of BSCVA remained unchanged in 17 cases, 1 case lost 2 lines, 1 case lost 1 line, 22 cases gained 1 line, and 3 cases gained 2 lines of visual acuity (Figure 11).

Re-treatment rate is 2.2% in the Group 1 compared to 8.2% in Group 2. The difference is highly significant (*p* < 0.01).

## 4. Discussion

The rotational eye-tracker system has the ability not only to correct the rotation of an eye due to a change of the body position but also the ability to correct cyclotoric movements intra-operatively. This intra-operative cyclotorsion can occur, for example, due to a loss of fixation of the patient, excitement, or small head rotations. To ensure accurate pulse position during treatment the recognition process is continuously active during laser ablation. These images are continuously compared to the reference image, which is the last image of the initial recognition process. For each image, the quality factor and rotation angle is calculated and the remaining pulse list is rotated to the actual valid rotation angle. Therefore, even if intra-operative cyclotorsions occur, the dynamic application of the iris recognition process and the following correction ensure accurate pulse positioning during the treatment. During laser treatment, the images of the eye can lose contrast, for example due to dehydrating of the corneal surface, consequently, the difference between the reference image and the actual real time image is increased. In this case, the software is capable of recording a new reference image with the last valid rotation angle and continues to track the rotation angle using the new reference image. In our study, a highly significant difference was observed between the postoperative cylindric correction with and without a cyclotorsion control. The mean cyclotorsion was 3.75° with a standard deviation of 3.1°. The total range of torsion was from −14.9° to +12.6°. If a cylindric refraction of 4 D should be corrected with a cyclorotation of 10° without a cyclotorsion control and a correction error of 1D will be expected calculated mathematically with vector analysis. It has been shown in other studies that 50%—and in some case up to 65%—of the population has a cyclotorsion difference between standing and horizontal positions of more than 2° [7,9,10]. The cyclorotation can increase in individuals even up to 17° [7,11]. This confirms that the cyclotorsion control is a very important tool regarding the clinical outcome. In Group 2 we detected one eye which lost one line and another eye that lost two lines of visual acuity. In our opinion, the higher grade of astigmatism error in Group 2 leads to higher aberrations and, consecutively, to a decrease of the best-corrected visual acuity in these specific cases. Our results show good agreement with other published literature [7,12,13,14]. Different studies investigated the amount of cyclotorsion of eyes during LASIK treatments. Swami et al. [9] marked 240 eyes preoperatively while the patients were seated. Immediately before beginning the treatment, they measured the rotational misalignment of the eyes on the supine patients and found a mean cyclotorsion of 4.1° (±3.7°) while 8% of the eyes showed a deviation greater than 10°. Ciccio et al. [7] repeated the measurements, marking the horizontal axis of 1019 eyes prior to wavefront measurements, and detected the mark on the supine patient under the laser. The angle of misalignment was measured and analyzed whereby a mean rotational angle of the eyes from seated to supine patient of 4.05° (±2.9°) was observed. Moreover, they found a difference between left eyes and right eyes in the predominant trend of the rotation with 46% of the patients showing bilateral excyclotorsion and only 1.7% displaying bilateral incyclotorsion.

As already mentioned, it is well known that iris patterns are unique properties of an individual (such as finger prints) and the probability of finding two objects with the same iris pattern is extremely low. The challenge is to utilize this fact in a way that the realization provides an accurate determination of the special alignment of the unique pattern when compared to a reference image of the same eye. The strategy to fulfil these criteria is developed by means of using a sophisticated approach to analyze the complex iris structures and to transform this information into a digital code of the iris. This code should be sufficiently unique but still compact enough to provide a realistic processing time according to the desired application.

A central iris recognition and comparison module is used in all the applications mentioned. This module is the main tool in the process of identification of the iris patterns as well as in the determination of the rotational misalignment between two specific images. The initial point for an iris recognition process is always the availability of two iris images that can be compared to each other. Depending on whether or not they belong to the same eye, the key question is whether there is a measurable rotational angle between the two images.

The study was approved by the local institutional review board (Kantonalethikkommission Nr 2006/11, Aargau) and adhered to the tenets of the Declaration of Helsinki.

## 5. Conclusions

In this study, we present the results of 88 treatment sessions, 44 with and 44 without a cyclorotational eye tracker system. There was no significant difference of clinical outcome regarding the spherical equivalent, however there was a highly significant difference regarding the cylinder treatment. The advantage of iris recognition ensures a much better laser spot position during the surgery while it is not always the case in an only X/Y eyetracker control procedure. The dynamic cyclorotational eye tracker system leads to a high level of cylinder control during corneal refractive surgery with the Excimer laser. Therefore, the proposed system used for LASIK results in highly predictable refraction quality with significantly less postoperative re-treatments.
